# Supplementary material for: Photoacoustic and Magnetic Resonance Imaging of Hybrid Manganese Dioxide-Coated Ultra-Small NaGdF4 Nanoparticles for Spatiotemporal Modulation of Hypoxia in Head and Neck Cancer
Source: Cancers (Basel). 2020 Nov 6;12(11):3294. doi: 10.3390/cancers12113294 (PMC7694772; doi:10.3390/cancers12113294)
Supplement: Supplementary file 1 [file cancers-12-03294-s001.pdf]

*cancers*

# Supplementary Materials:

# Photoacoustic and Magnetic Resonance Imaging of Hybrid Manganese Dioxide Coated Ultra-small $NaGdF_4$ Nanoparticles for Spatiotemporal Modulation of Hypoxia in Head and Neck Cancer

**Laurie. J. Rich[1,+], Jossana A. Damasco[2,§], Julia C. Bulmahn[2], Hilliard L. Kutscher[2,3,4], Paras N. Prasad[2,*], and Mukund Seshadri[1,5,*]**

$$(1)\ 2MnO_2 + H_2O_2 \rightarrow 2MnOOH + O_2$$

$$(2)\ 2MnOOH + 4H^+ + H_2O_2 \rightarrow 2Mn^{2+} + 4H_2O + O_2$$

$$(3)\ 2MnOOH + 2H^+ \rightarrow MnO_2 + Mn^{2+} + 2H_2O$$

$$(4)\ MnO_2 + H_2O_2 + 2H^+ \rightarrow O_2 + Mn^{2+} + 2H_2O$$

**Figure 1. Reaction scheme of $MnO_2$ with $H_2O$ under acidic conditions[1-4].** Under acidic conditions, the $MnO_2$ can undergo redox reaction with $H_2O_2$ (1) to form $H_2O$, $O_2$ and $Mn^{2+}$ (1-3). The MnOOH facilitates the decomposition of $H_2O_2$ to $H_2O$ and $O_2$ as the $Mn^{3+}$ is further reduced to $Mn^{2+}$ (2). MnOOH can also undergo disproportionation reaction to reform $MnO_2$, and also produce $Mn^{2+}$ and $H_2O$ (3). The overall reaction is given in (4).

[1]Luo XL, Xu JJ, Zhao W, Chen HY. A novel glucose ENFET based on the special reactivity of MnO2 nanoparticles. Biosens Bioelectron. 2004 May 15;19(10):1295-300. doi: 10.1016/j.bios.2003.11.019. PMID: 15046762.

[2]Fan W, Bu W, Shen B, He Q, Cui Z, Liu Y, Zheng X, Zhao K, Shi J. Intelligent MnO2 Nanosheets Anchored with Upconversion Nanoprobes for Concurrent pH-/H2O2-Responsive UCL Imaging and Oxygen-Elevated Synergetic Therapy. Adv Mater. 2015 Jul 22;27(28):4155-61. doi: 10.1002/adma.201405141. Epub 2015 Jun 8. PMID: 26058562.

[3]Chen Q, Feng L, Liu J, Zhu W, Dong Z, Wu Y, Liu Z. Intelligent Albumin-MnO2 Nanoparticles as pH-/H2 O2 -Responsive Dissociable Nanocarriers to Modulate Tumor Hypoxia for Effective Combination Therapy. Adv Mater. 2016 Sep;28(33):7129-36. doi: 10.1002/adma.201601902. Epub 2016 Jun 10. Erratum in: Adv Mater. 2018 Feb;30(8): PMID: 27283434.

[4]Long JW, Rhodes CP, Young AL, Rolison DR. Ultrathin, Protective Coatings of Poly(o-phenylenediamine) as Electrochemical Proton Gates: Making Mesoporous MnO2 Nanoarchitectures Stable in Acid Electrolytes. Nano Letters 2003 3 (8), 1155-1161. DOI: 10.1021/nl0343598

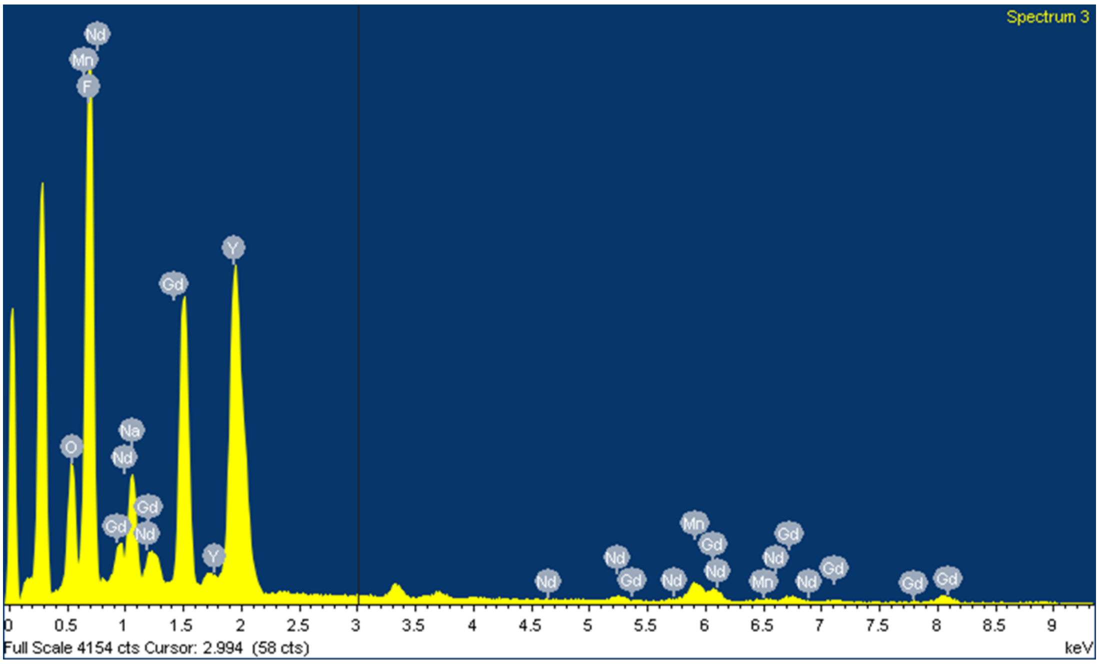


| Element | Atomic % |
|---|---|
| O | 13.95 |
| F | 68.06 |
| Na | 4.99 |
| Mn | 3.04 |
| Y | 8.68 |
| Nd | 0.3 |
| Gd | 0.98 |

**Figure 2.** Elemental analysis using energy dispersive x-ray analysis confirmed all the elements (Y, Nd, Gd) in the synthesis were successfully incorporated in the usNP. Presence of Mn verified the successful coating of MnO2. The 1:3 ratio of Gd to Mn supports the findings of the ICP-OES analysis.

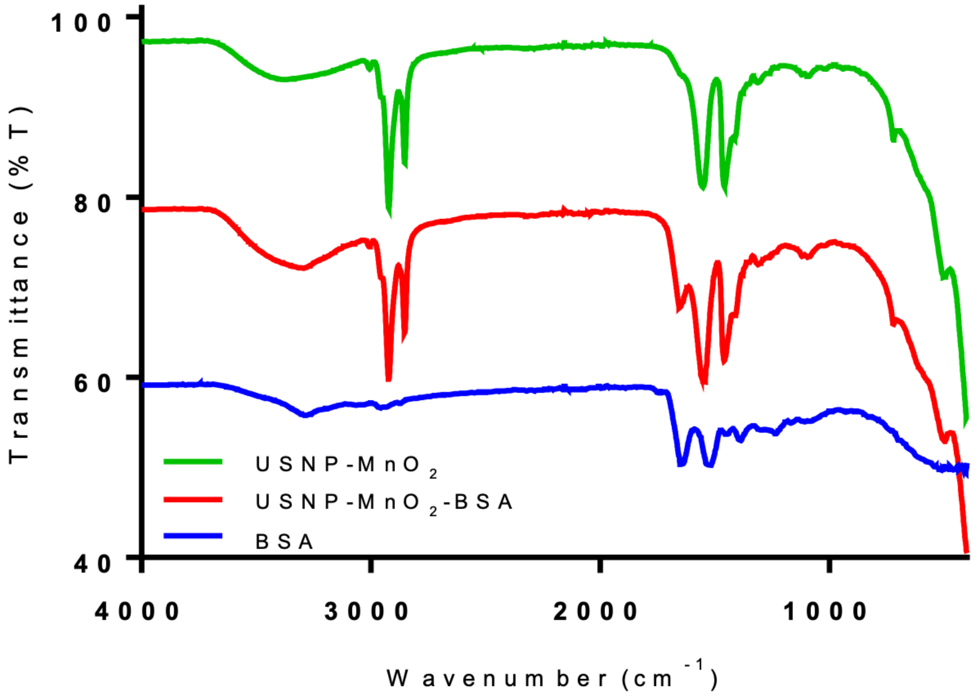


**Figure 3.** FTIR revealed presence of the ~1635 cm-1 peak, characteristic of the amide I band of BSA, in the spectra of BSA-usNP-MnO2 indicating the successful formation of BSA on the surface of usNp-MnO2[5].

[5]Yu, S., Laromaine, A. & Roig, A. Enhanced stability of superparamagnetic iron oxide nanoparticles in biological media using a pH adjusted-BSA adsorption protocol. J Nanopart Res 16, 2484 (2014). https://doi.org/10.1007/s11051-014-2484-1

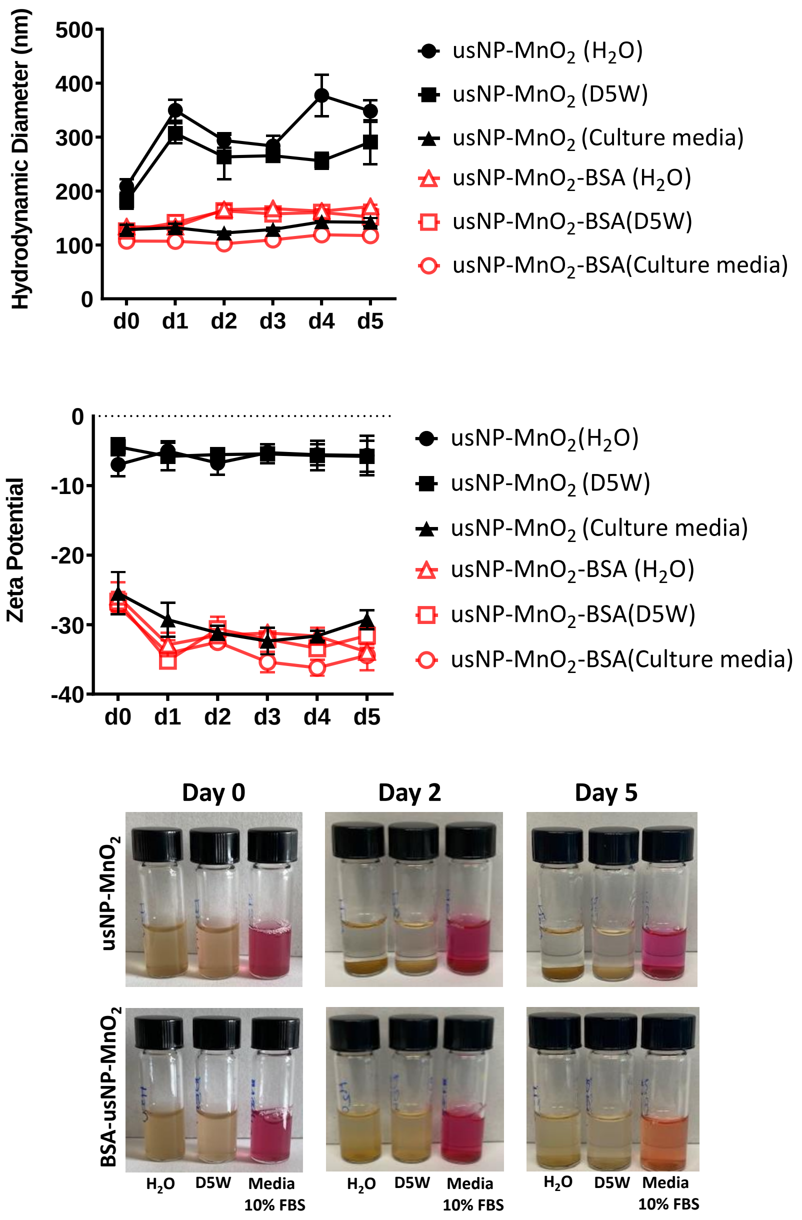


**Figure 4. Stability study of usNP-MnO₂ and BSA-usNP-MnO₂.** The hydrodynamic diameter (**A**) measured from dynamic light scattering (% Number) and the zeta potential (**B**) showed improved stability due to the electrostatic repulsion provided by the BSA coating. The zeta potential decreased from > -10 to ~-30 with the coating of BSA. Photographs (**C**) of the usNP-MnO2 and BSA-usNP-MnO2 suspended in different solvents showed the freshly prepared nanoparticles were stable in all solvents. Over time, aggregations on the bottom were observed due to the large size of the nanostructures and desorption of BSA from the surface.

[6]Wu, F. S.; Yue, L. L.; Cheng, K.; Chen, J.; Wong, K. L.; Wong, W. K.; Zhu, X. J. Facile Preparation of Phthalocyanine-Based Nanodots for Photoacoustic Imaging and Photothermal Cancer Therapy In Vivo. ACS Biomater Sci Eng 2020, 6, 5230-5239.

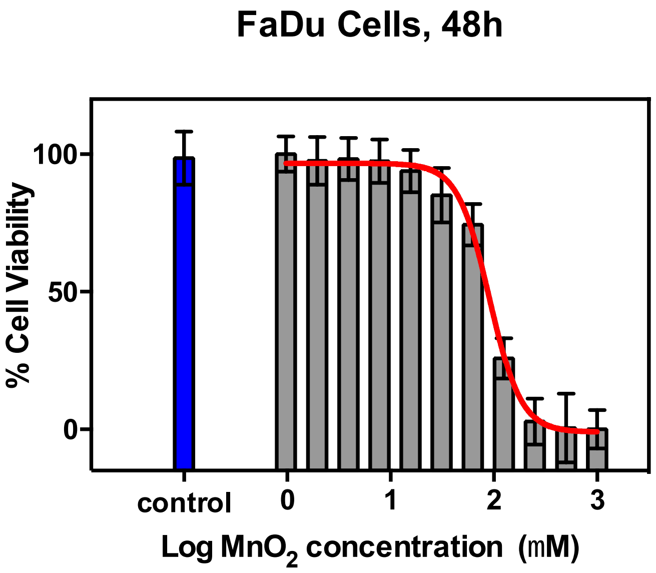


**Figure 5. Cell viability assay of BSA-usNP-$MnO_2$.** Viability of FaDu cancer cells (10,000 cells/well) exposed to increasing concentrations of BSA-usNP-$MnO_2$ (0, 0.10, 0.97, 1.95, 3.91, 7.81, 15.6, 31.2, 62.5, 125, 250, 500, 1000 μM MnO2) for 48 hours. IC50 was determined to be at 90uM $MnO_2$ from the 3-(4,5-dimethylthiazol-2-yl)-2,5-diphenyltetrazolium bromide (MTT) assay.

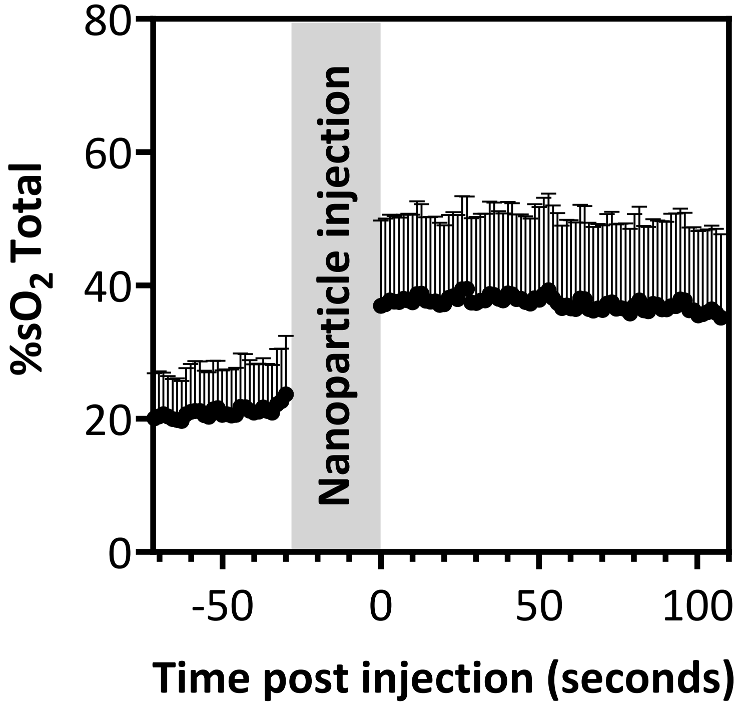


**Figure 6. Dynamic time course of PAI measurements following IV injection of usNP-$MnO_2$ nanoparticles.** These %s$O_2$ measurements suggest that tumor oxygenation levels increase rapidly and stay elevated over several minutes.
